# Wormpath: searching for molecular interaction networks in *Caenorhabditis elegans*

**DOI:** 10.1186/s13029-015-0034-6

**Published:** 2015-04-02

**Authors:** Peter Frommolt, Björn Schumacher

**Affiliations:** CECAD Research Center, University of Cologne, Joseph-Stelzmann-Str. 26, 50931 Cologne, Germany; Cologne Center for Genomics, University of Cologne, Cologne, Germany; Systems Biology of Aging Cologne (SyBACol), University of Cologne, Cologne, Germany; Institute for Genome Stability in Aging and Disease, Medical Faculty, University of Cologne, Cologne, Germany; Center for Molecular Medicine Cologne (CMMC), University of Cologne, Cologne, Germany

**Keywords:** Molecular interaction networks, Gene expression, Wormbase, Web application

## Abstract

**Background:**

High-throughput transcriptional profiling using Next-Generation Sequencing (RNA-Seq) or microarray technology have become standard tools in molecular biology. Successful investigations of gene regulatory mechanisms from these data typically employ mathematical models of biological networks.

**Results:**

We have developed *Wormpath*, a software for molecular network discovery which operates on the genetic and physical interaction data of the Wormbase, a comprehensive resource of molecular data on *Caenorhabditis elegans*. We use Wormpath to show that the insulin/insulin-like growth factor signalling (IIS) pathway responds to UV-induced DNA damage during development.

**Conclusions:**

Our software provides highly facilitated access to *C. elegans* interaction data and is capable of identifying essential molecular networks within a list of differentially expressed genes.

**Electronic supplementary material:**

The online version of this article (doi:10.1186/s13029-015-0034-6) contains supplementary material, which is available to authorized users.

## Background

Genome-wide expression profiling is achieved by high-throughput sequencing (RNA-Seq) or by expression microarrays. An initial analysis of the resulting data is typically achieved by sorting the entirety of all genes by statistical evidence for differential expression compared to a control condition. For downstream analysis, statistics about the functional properties enriched in a set of differentially expressed genes are needed as well as information on mutual dependencies between genes or larger molecular networks. The nematode worm *Caenorhabditis elegans* is a widely used model organism in developmental biology and genetics [1] and subject to multiple studies based on high-throughput gene expression data (e.g. [2]). In this paper, we describe *Wormpath*, a software enabling the discovery of complex networks among a set of differentially expressed genes which is specifically optimized for use in *C. elegans* studies. Recently, we used Wormpath as part of a larger study on the DNA damage response [3].

Since its inception in 1998, the Wormbase [4] has emerged as a widely used repository for genomic information on *C. elegans* and other nematodes, including comprehensive knowledge on previously reported molecular interactions. These data comprise genetic as well as physical interactions and are based on a large variety of experimental methods such as yeast one-, two-, and three-hybrid assays as well as RNAi in mutant backgrounds. The interactions are both manually and automatically curated with an emphasis on including large-scale data sets [5]. Wormpath takes advantage of this resource to search for networks between members of a user-provided list of differentially expressed genes. For any of these networks, the software reports statistical measures, graphical plots and functional annotation by GO terms.

Other tools for molecular network discovery from high-throughput data include *Cytoscape* [6] or web-based software like the *Pathway Projector* [7] – a selection of related tools was reviewed by Thomas and Bonchev [8]. As the aforementioned software programs, Wormpath is freely available but, in contrast to the others, it is specifically tailored for analyses in *C. elegans* and closely interacts with the comprehensive knowledge in the Wormbase. By adopting our software, researchers from the worm field can now use extensive information on molecular interactions of *C. elegans* without additional efforts like local software installation or database maintenance.

### Implementation

Our software ignores interactions which are only computationally predicted, e.g. from orthologous interactions, but not experimentally confirmed in C. elegans. Furthermore, we call an *indirect* interaction a pair of interactions that connect two nodes representing genes from the list of differentially expressed genes to an additional node that acts as a *linker gene* which is not differentially expressed. This reflects the fact that a key gene might have been missed, for instance due to technical reasons. Given a list $\mathbb{L}$ of differentially expressed genes, our approach relies on a large basic graph **G** = (*V*, *E*) with a set *V* of vertices and a set *E* of edges, representing the topology of the network between members of $\mathbb{L}$. Genes are represented by nodes in *V* and interactions between genes or their proteins are represented by edges in *E* labeled as either *direct* or *indirect*.

Searching the graph **G** for networks is closely related to the problem of finding the *connected components* of **G** defined as all connected subgraphs which are not subgraphs of any larger connected graph, where a graph is called *connected* if each pair of its nodes is connected by a path. Searching the connected components is a classical problem for which a typical solution is formed e.g., by a depth-first search algorithm [9]. Members of larger candidate gene sets often form highly interconnected networks because it is likely that many of them are players in the same biological processes. Thus, the connected components of the basic graph easily become very large themselves. To avoid this, our approach is controlled by an iteration depth *D* defining the number of allowed steps from a start node. The software localizes the first gene of $\mathbb{L}$ on **G** and searches all *d*-neighbours, i.e. all nodes that are reachable in at most *d*∈{*1*, …, *D*} steps from that position, with both *direct* and *indirect* interactions taken into account. After replacing the *indirect* interactions by the two respective interactions plus a new node for the linker gene, the software builds a new graph **N** = (*V*_*N*_, *E*_*N*_) composed of only the *d*-neighbours and the edges between them. This procedure is repeated with the remaining genes of the list. Statistical significance of the networks is assessed and the results are sorted according to their network scores (see below). The algorithm is then repeated with *D* replaced by *D-1*, …, *1* to generate additional networks which are of smaller complexity.

To assess the statistical features of a network **N** in the results list, **N** is assigned a score defined as the average number of citations supporting its edges, providing a measure of the strength of experimental evidence for **N**. To test whether this network score is larger than expected by chance alone, the score is re-calculated for a given number of randomly simulated networks and a *p*-value is calculated as the fraction of those simulated scores which are at least as large as the original score.

Furthermore, the *induced sub-network* composed of all *1*-neighbours of **N** is given by **N**^**1**^ = {(*v*, *e*) ∈ **G** | v ∈ *V*; ∃ ṽ∈*V*_***N***_: *d*(*v*, *ṽ*) = 1}. We consider the null hypothesis *H*_*0*_ that the proportion of genes that are elements of **N**^**1**^ is equal in $\mathbb{L}$ and in the entirety of all *C. elegans* genes. If *H*_*0*_ can be rejected, we conclude that the members of **N**^**1**^ are significantly over-represented in $\mathbb{L}$ and the presence of **N** in $\mathbb{L}$ is unlikely to have occurred by chance alone.

If the database contains a total number of *N* genes, the number *X* of members of **N**^**1**^ contained in $\mathbb{L}$ is a hypergeometrically distributed random variable with parameters *N*, |**N**^**1**^|, and |$\mathbb{L}$| and therefore has got the probability density function f^X^ with

$$ {f}^X\left(\mathrm{k}\right):=\mathrm{P}\left(\mathrm{X}=\mathrm{x}\right)=\frac{\left(\begin{array}{c}\hfill N-\left|{\boldsymbol{N}}^1\right|\hfill \\ {}\hfill \left|\mathbb{L}\right|-k\hfill \end{array}\right)\left(\begin{array}{c}\hfill \left|{\boldsymbol{N}}^1\right|\hfill \\ {}\hfill k\hfill \end{array}\right)}{\left(\begin{array}{c}\hfill N\hfill \\ {}\hfill \left|\mathbb{L}\right|\hfill \end{array}\right)} $$

From this, the *p*-value for **N** can be calculated by $p={\displaystyle {\sum}_{\tilde{k}=k}^n{f}^X\left(\tilde{k}\right)}$. If this *p*-value is sufficiently small, *H*_*0*_ can be rejected at the predefined significance level.

Our approach to statistical assessment of the networks resulting from a Wormpath analysis covers two distinct scientific aspects. The network score and its corresponding score-based *p*-value provide a measure of the strength of experimental evidence for the interactions involved in the network. On the other hand, the result from the second significance test termed the *list-based p*-value tells us to what extent differentially expressed genes are over-represented in the network compared to the entire genome. In some cases, these two approaches can lead to opposite results for the same network (see Results and Discussion); however, their combined information draws a broader picture of the statistical features of the respective molecular network than one of them alone.

The Wormpath software itself has been implemented using Perl/CGI and takes advantage of GraphViz [10] to layout the networks and generate the graphical output. Users can browse their results online or save a compressed version of the output to their local computer. The computation is based on a local database synchronized with the Wormbase using the AcePerl API. The source code of Wormpath as of version 1.0.4 is provided as a supplement to this manuscript (Additional file 1).

## Results and discussion

**Figure 1:**
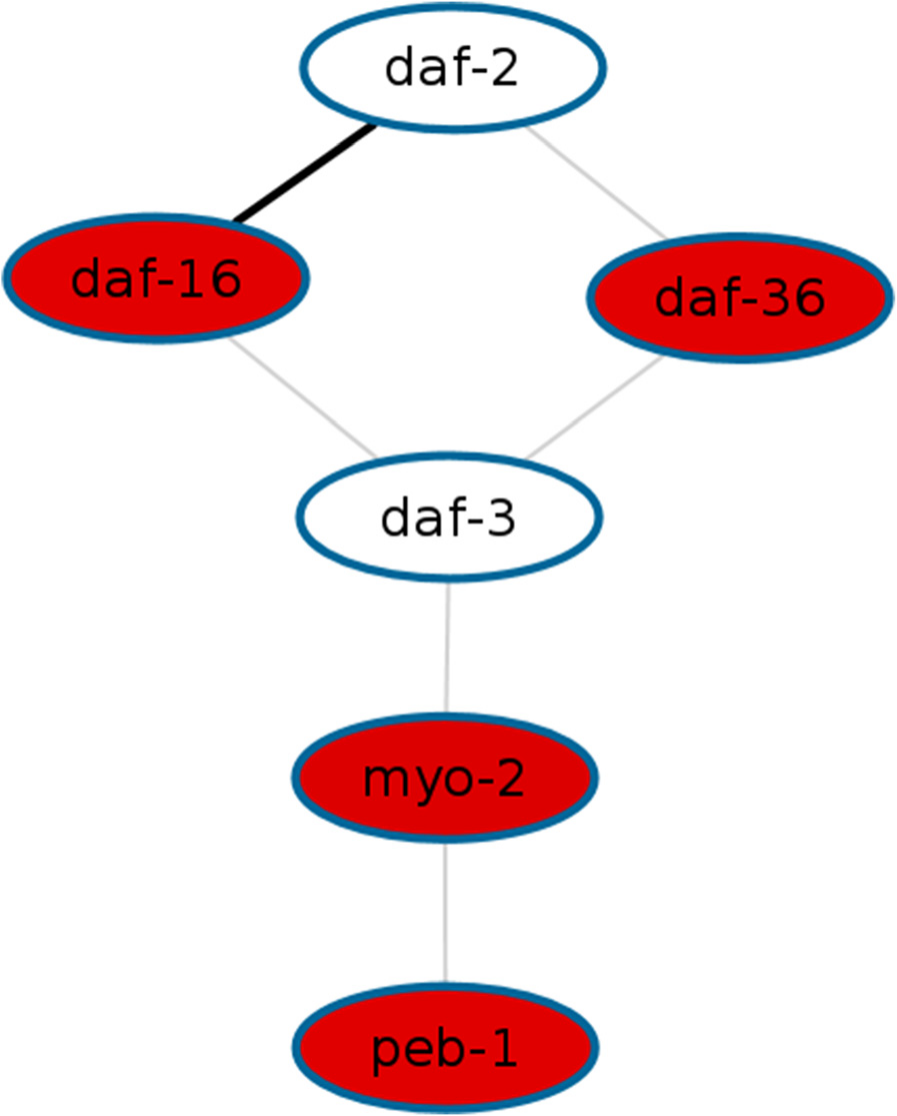
**Top-scoring network among genes altered by UV radiation.** In an analysis of a set of 1349 genes differentially expressed upon UV radiation, Wormpath revealed 25 significantly enriched networks. The network shown exhibits the strongest statistical evidence among all results (score 23.33; *p* < 0.0001). Red nodes represent induced genes whereas white nodes represent unchanged genes. Any edge denotes an interaction between the two genes, where a grey line indicates that the interaction has been described in only one publication and the bold line between *daf-16* and *daf-2* indicates that this interaction has been described multiple times in the literature (135 papers).

To show the potential of our approach for molecular network discovery, we re-analyzed microarray data from our recent study in which we reported that genes responding to DNA damage during development are enriched in the insulin/insulin-like growth factor signalling (IIS) pathway [3]. Transcriptional profiling of *C. elegans* L1 larvae after 6 h under UV irradiation at 60 mJ/cm^−2^ resulted in a list of 1349 differentially expressed genes (*p* < 0.01; fold change > ±1.5; Additional file 2: Table S1, online). Using iteration depth *D* = 1, Wormpath identifies a list of 25 molecular interaction networks significantly enriched among this set of genes (*p* < 0.01; Table 1). The *p*-values based on the network score and those based on the test for enrichment of differentially expressed genes represent two different statistical aspects of the resulting networks and the results for the two methods are opposite for some of them (Table 1). The IIS genes *daf-16*/*FoxO* and *daf-2*/*Igf1* are part of 10 (*daf-16*) and 4 (*daf-2*) out of these 25 networks, indicating that they play a central regulatory role among the genes altered upon UV irradiation during development. The highest-scoring network (score 23.33; *p* < 0.0001) comprises both *daf-16* and *daf-2* (Figure 1). Wormpath links this network to a DAVID [11] analysis which reveals that *dauer larval development* is among the GO terms enriched for the genes involved, indicating that they do in fact respond to DNA damage.

**Table 1:** **Networks identified in a Wormpath analysis of 1349 genes regulated upon UV radiation**

	**Genes involved**	**Nodes**	**Edges**	**Score**	**p-value (score)**	**p-value (list)**
**1**	daf-3, peb-1, myo-2, daf-36, daf-16, daf-2	6	6	23.33	0.0006	<0.0001
**2**	cav-1, sur-5, sma-6, lin-28, let-653, kin-15, unc-52, let-60, unc-6, ces-1, col-48, glp-1, daf-2, daf-12, F40F4.6, sqt-1, daf-16, kin-9	18	19	8.58	0.001	<0.0001
**3**	sbds-1, daf-3, ins-7, F26A1.9, acs-20, nhr-49, lys-7, daf-16, C26B9.3, mtl-1, agmo-1, acs-2, npp-9, myo-2, C29F9.2, daf-2, gei-7, C14A4.9, cyp-42A1, let-653, daf-36, nstp-3, T22B11.2	23	26	6.58	0.0018	<0.0001
**4**	cyp-42A1, daf-16, ugt-62, nhr-49, gei-7, daf-36, npp-9, lys-7, C14A4.9, C26B9.3, daf-2, F40F4.6, sbds-1, egl-15, let-756, acs-2, acs-20, ins-7, nstp-3, mtl-1, C29F9.2, agmo-1, T22B11.2, T24C12.4, daf-3, F26A1.9, ZK418.7, let-653	28	31	5.68	0.0018	<0.0001
**5**	cav-1, lon-3, sqt-1, unc-52, unc-6, glp-1, kin-15, F40F4.6, sqt-3, let-60, rol-6	11	12	5.58	0.009	<0.0001
**6**	F26A1.9, acs-2, daf-2, C29F9.2, lys-7, daf-36, agmo-1, nhr-49, sur-5, daf-3, let-653, vab-1, C14A4.9, unc-6, daf-16, ces-1, daf-12, cyp-42A1, mtl-1, C26B9.3, nstp-3, cav-1, let-60, T22B11.2, npp-9, nhr-8, kin-9, sbds-1, ins-7, acs-20, gei-7, qua-1	32	36	5.17	0.0042	<0.0001
**7**	unc-2, nhr-49, C14A4.9, let-653, daf-16, C29F9.2, unc-25, mrp-1, agmo-1, let-60, sbds-1, daf-2, fasn-1, daf-11, nsy-4, cyp-42A1, cav-1, lin-28, daf-12, daf-7, ins-7, mtl-1, C26B9.3, myo-2, F26A1.9, daf-3, acs-20, sir-2.1, lys-7, T22B11.2, abu-11, nstp-3, acs-2, daf-36, daf-1, gei-7, npp-9	37	42	4.81	0.003	<0.0001
**8**	lin-14, daf-16, daf-12, cav-1, col-17, ins-33, lin-46, lin-28	8	9	2.67	0.0814	<0.0001
**9**	lim-6, daf-16, unc-25, zig-2, fasn-1, daf-7	6	5	1.80	0.3028	<0.0001
**10**	daf-1, daf-11, mrp-1, fasn-1, unc-25, daf-16, daf-7	7	7	1.71	0.3576	<0.0001
**11**	sur-5, let-60, ces-1, F56D2.3, kin-9, crb-1, cav-1, let-653, phg-1, atl-1, clk-2, unc-6	12	11	1.64	0.361	<0.0001
**12**	ces-1, unc-6, let-60, ced-11, ced-3, kin-9, sur-5, let-653, cav-1	9	8	1.62	0.418	<0.0001
**13**	glp-1, cav-1, kin-15, F40F4.6, sqt-1	5	4	1.50	0.4766	<0.0001
**14**	peb-1, lin-31, sur-5, lin-45, kin-9, let-60, ces-1, unc-6, let-653, cav-1	10	9	1.44	0.5824	<0.0001
**15**	sur-5, egl-30, sqt-1, cav-1, unc-6, glp-1, ces-1, unc-52, gtl-1, mig-17, kin-9, let-653, egl-8, let-60, lev-11	15	15	1.40	0.6734	<0.0001
**16**	F40F4.6, ugt-62, glp-1, T24C12.4, let-756, npp-9, sqt-1, kin-15, cav-1	9	8	1.25	0.76	<0.0001
**17**	let-23, che-14, lin-45, sur-5, lin-26, dsl-5, peb-1, ZK418.7, lin-31	9	8	1.00	>0.9999	0.0018
**18**	let-23, lin-31, dsl-5, ZK418.7, col-48, bar-1	6	5	1.00	>0.9999	<0.0001
**19**	egl-15, dsl-5, ZK418.7, npp-9, lin-31, let-23	6	5	1.00	>0.9999	<0.0001
**20**	acn-1, mlt-9, nhr-23, qua-1, nhr-25	5	4	1.00	>0.9999	0.0005
**21**	ptr-16, ptr-1, ptr-6, ptr-10	4	4	1.00	>0.9999	<0.0001
**22**	sma-6, cav-1, bar-1, dsl-5, col-48	5	4	1.00	>0.9999	<0.0001
**23**	dpy-13, bli-4, dpy-5, dpy-6	4	3	1.00	>0.9999	<0.0001
**24**	nsy-4, daf-16, unc-2	3	2	1.00	>0.9999	0.0037
**25**	sym-5, sym-1	2	1	1.00	>0.9999	0.0046

Although *daf-2*, the *C. elegans* orthologue of the human insulin-like growth factor 1 (*Igf1*) receptor, is not differentially expressed upon UV irradiation, the software recognizes that *daf-16* as well as *daf-2* are central players. This illustrates the effectiveness of using linker genes as in our approach. Regarding the statistical evaluation, the largest network scores are achieved by networks with strong experimental evidence for their interactions. In our analysis of the 1349 UV-responsive genes, the network score is therefore maximized by the smallest network among those which contain the *daf-16*/*daf-2* interaction given that the negative regulation of *daf-16* activity downstream of *daf-2* has been described multiple times in the literature. In our recent study [3], however, we have reported the network with the largest number of interactions as this gives the most comprehensive selection of candidate genes related to the IIS pathway. In contrast, the network score defined in this paper is rather useful to assess the strength of scientific evidence for a particular network.

A summary of the features of Wormpath compared to those of other tools is given in Table 2. Among the softwares listed, Wormpath is the only one that is easily accessible to benchside biologists *and* can be used to systematically search a list of candidate genes for molecular networks in *C. elegans*. For instance, the usage of Cytoscape requires at least basic knowledge on the structure and sources of the interaction database that is used and mapping your own data to the resulting network requires deeper software skills. Also, Cytoscape itself does not provide any functionality to extract or statistically evaluate existing sub-networks. The Pathway Projector, on the other hand, can use candidate genes identified from experimental data only to highlight them on existing KEGG pathways in *C. elegans*, whereas a systematic search and assessment of networks within the candidate genes is not provided. Finally, Ingenuity Pathway Analysis is a very powerful software with a comprehensive back-end database, but it is subject to commercial licensing and supports the analysis of networks in *C. elegans* data only by homology mapping. In contrast to these softwares, Wormpath greatly facilitates access to the Wormbase interaction database and uses this rich resource to build molecular networks from a list of candidate genes uploaded by the user. In addition, the software is available free of charge and the design of its interface is very intuitive and easy to use.

**Table 2:** **Features of Wormpath compared to other softwares**

	**Wormpath**	**Cytoscape**	**Pathway projector**	**Ingenuity pathway analysis**
**Species**	C. elegans	Generic	Multiple	Human/Mouse/Rat
**Implementation**	Web application/Perl, CGI	Desktop application/Java	Web application/Google Maps API	Web application/Java
**Database**	Wormbase	Generic	KEGG	Undisclosed
**Scope of analyses**	Networks/Functional annotation	Networks/Multiple add-ons	Search details on pathways/Map experimental data	Networks/Functional annotation
**Target audience**	Bench scientists	Computational biologists	Bench scientists	Bench scientists
**Statistics**	Scores, significance tests	Add-ons required	None	Scores, significance tests
**License**	GPL	LGPL	KEGG license required	Commercial

Among the goals of Wormpath’s future development is the application of its methodology also to data from other model organisms such as *Drosophila melanogaster* (fruit fly) and *Saccharomyces cerevisiae* (yeast). Furthermore, the interaction database used by the software will be enriched with interaction data from other sources including ChIP-Seq data from the modENCODE project [12] and miRNA target gene prediction algorithms (reviewed by [13]).

## Conclusions

We have shown that the Wormpath software is generally suitable for the discovery of networks composed of previously described molecular interactions of the worm. Our software facilitates downstream analysis of experiments based on high-throughput transcriptional profiling and enables an easy approach to network biology in *C. elegans*.

## Availability and requirements

**Project name:** Wormpath

**Project home page:**http://bifacility.uni-koeln.de/wormpath

**Operating system:** Browser-accessible web service, platform-independent

**Programming language:** Perl/CGI

**Other requirements:** None

**License:** GPL version 3

**Restrictions to use by non-academics:** The software is accessible without any restrictions.

## Additional files

Additional file 1:
**Source code of the Wormpath software as of version 1.0.4 (February 2015).**
Additional file 2: Table S1. List of 1349 genes differentially expressed upon UV radiation. The file is suitable as an input file for the Wormpath software.

## Abbreviations

RNA-Seq: RNA sequencing
UV: Ultra violet
IIS: Insulin/insulin-like signaling
CGI: Common gateway interface
GO: Gene ontology
